# Electrical conductivity of nutrient solution influenced photosynthesis, quality, and antioxidant enzyme activity of pakchoi (*Brassica campestris* L. ssp. *Chinensis*) in a hydroponic system

**DOI:** 10.1371/journal.pone.0202090

**Published:** 2018-08-29

**Authors:** Xiaotao Ding, Yuping Jiang, Hong Zhao, Doudou Guo, Lizhong He, Fuguang Liu, Qiang Zhou, Dilip Nandwani, Dafeng Hui, Jizhu Yu

**Affiliations:** 1 Shanghai Dushi Green Engineering Co., Ltd. Shanghai Key Lab of Protected Horticultural Technology, Shanghai Academy of Agricultural Sciences, Shanghai, China; 2 School of Agriculture and Biology, Shanghai Jiaotong University, Shanghai, China; 3 Department of Agricultural and Environmental Sciences, Tennessee State University, Nashville, Tennessee, United States of America; 4 Department of Biological Sciences, Tennessee State University, Nashville, Tennessee, United States of America; Northwest Agriculture and Forestry University, CHINA

## Abstract

To find an electrical conductivity (EC) in the nutrient solution used for pakchoi (*Brassica campestris* L. ssp. *Chinensis*) cultivation that optimizes the plant’s physiology, growth, and quality, we conducted an experiment with eight EC treatments (from EC0 to EC9.6) in a hydroponic production system (i.e. soilless culture) under greenhouse condition in Shanghai, China. Plants biomass production, leaf photosynthesis, vegetable quality variables, tissue nitrate and nitrite contents, and antioxidant enzyme activities were measured. The results showed that very high (EC9.6) or low EC (EC0-0.6) treatments clearly decreased plants fresh weight (FW) and dry weight (DW), leaf size, leaf water content, leaf net photosynthetic rate (*P*_*n*_), stomatal conductance (*G*_*s*_), transpiration rate (*T*_*r*_), and taste score. Nitrite content, and antioxidant enzyme activities were low in medium EC treatments (EC1.8 and EC2.4), but high in very high or low EC treatments. Leaf relative chlorophyll, ascorbic acid, and nitrate contents increased gradually from low EC to high EC treatments, while crude fiber and soluble sugar contents decreased. Based on growth and quality criteria, the optimal EC treatment would be EC1.8 or EC2.4 for pakchoi in the hydroponic production system. Too high or too low EC would induce nutrient stress, enhance plant antioxidant enzyme activities, and suppress pakchoi growth and quality.

## Introduction

Plant physiology, growth, and development are closely associated with the environmental conditions and nutrient supply [1, 2]. Optimization of the nutrient application to plants is fundamental to improve crop production, especially for fast-growing leaf vegetables [3, 4]. Inadequate management of nutrient solution such as the use of a too high or a too low concentration of the nutrient solution, or an imbalanced ion composition could inhibit plant growth due to either toxicity or nutrient-induced deficiency [5].

The electrical conductivity (EC) is an index of salt concentration and an indicator of electrolyte concentration of the solution. EC of the nutrient solution is related to the amount of ions available to plants in the root zone [6]. The optimal EC is crop specific, and depends on environmental conditions [7, 8]. In general, higher EC hinders nutrient uptake by increasing the osmotic pressure of the nutrient solution, wastes nutrients, and the increases discharged of nutrients into the environment, resulting in environmental pollution. Lower EC may severely affect plant health and yield [2, 9].

Vegetables in the Brassicaceae family are an important food source in Asian countries such as China, Japan, and India, and in the European Union [10]. Pakchoi is a widely cultivated leafy vegetable in China. It provides phenolic compounds, vitamins, fibers, soluble sugars, minerals, fat, and carotenoids which included in human diets [11, 12]. As the consumption of pakchoi has been increasing during recent years, the safety and quality of pakchoi has become a major concern [13,14].

Several studies have been done on the effects of nutrient solution EC on leafy vegetable growth such as lettuce [9, 15, 16]. For example, Samarakoon et al. [9] found the best EC of the nutrient solution was 1.4 dS m^-1^ for lettuce under tropical greenhouse conditions (38.5°C). Raising the EC to 2 dS m^-1^ did not significantly increase leaf growth and yield. Albornoz et al. [17] revealed that applying of different nutrient solution concentrations (i.e. different EC) during day and night reduced NO_3_^−^ concentration in lettuce leaves without having much influence on leaf production. Albornoz and Lieth [18] demonstrated that over fertilization (high EC) limited lettuce productivity because of osmotic stress. But so far, few studies have been conducted to investigate the effects of EC on pakchoi growth and development, especially on the yield, quality, and the potential regulation mechanisms of pakchoi plants growing in different EC nutrient solutions.

The objective of this study was to assess the effect of different EC nutrient solutions on the growth, leaf photosynthesis, quality, and activities of antioxidant enzymes in pakchoi. In addition, we attempted to seek the optimal nutrient EC for both pakchoi growth and eating quality, and the underlying mechanisms of physiological changes of plants in too high or too low nutrient solution concentrations.

## Materials and methods

### Plant materials and experimental design

A cultivar of pakchoi (*Brassica campestris* L. ssp. *Chinensis* (L.)), Shanghaiqing, was used in this study. Pakchoi seeds were sown in Grodan blocks (2–4 seeds/ block, 10 cm x 10 cm x 6.5 cm) in a well heated greenhouse. Temperature in the greenhouse was maintained at about 20°C during the day and 10°C at night. Plants grew under natural light in the greenhouse. When the plant’s sixth true leaf was fully expanded, eight blocks of seedlings were transplanted into each plastic container (44 cm x 28 cm x 6.5 cm) for different EC treatments. The eight EC treatments in this study were arranged in a randomized complete block design. The experiment was replicated three times with one experimental unit (hydroponic system, eight plants) per treatment. The original nutrient solutions (A and B) were based on Hoagland’s solution (HS) with modifications and the nutrient concentrations were listed in Table 1. Nutrient solutions for the EC treatments were described below. Plants grew under the EC treatments for 20 days, and the largest leaves of each treatment were harvested. The samples were immediately frozen in liquid nitrogen and stored at -80°C for further analysis.

**Table 1:** Elements component in the original nutrient solutions in different tanks (A, B,).

A Tank				B Tank	
Macro-elements	kg/1000L	Micro-elements	g/1000L	Macro-elements	kg/1000L
KNO_3_	12.2	MnSO_4_·H_2_O	55.8	KNO_3_	32.0
Ca(NO_3_)_2_·4H_2_O	27.9	ZnSO_4_·7H_2_O	35.8	KH_2_PO_4_	13.6
5Ca(NO_3_)2·NH_4_NO_3_·10H_2_O	18.9	Na_2_B_4_O_7_·4H_2_O	118.9	K_2_SO_4_	1.1
EDPA-Fe (13%Fe)	1.0	CuSO_4_·5H_2_O	9.3	MgSO_4_·7H_2_O	12.3
		Na_2_MoO_4_·2H_2_O	6.0		

### Nutrient solution treatments

Portable Conductivity Meter (DDB-303A, Shanghai Leici) and pH tester (PHB-4, Shanghai Leici) were used to measure and set different EC treatments and pH value of the nutrient solutions. The different nutrient solutions were prepared with deionized water and soluble fertilizers (Shanghai Wintong Chemicals Co., Ltd.), based on original nutrient solutions. The eight different EC treatments included: 1) EC0, only deionized water; 2) EC0.3, diluted original nutrient solutions A and B with deionized water to 0.3 dS m^-1^ of EC; 3) EC0.6, diluted original nutrient solutions A and B with deionized water to 0.6 dS m^-1^ of EC; 4) EC1.2, diluted original nutrient solutions A and B with deionized water to 1.2 dS m^-1^ of EC; 5) EC1.8, diluted original nutrient solutions A and B with deionized water to 1.8 dS m^-1^ of EC; 6) EC2.4, diluted original nutrient solutions A and B with deionized water to 2.4 dS m^-1^ of EC; 7) EC4.8, diluted original nutrient solutions A and B with deionized water to 4.8 dS m^-1^ of EC; 8) EC9.6, diluted original nutrient solutions A and B with deionized water to 9.6 dS m^-1^ of EC. Every time the same amounts of A and B were used. We use hydrochloric acid (HCl) and sodium hydroxide (NaOH) to adjust pH of the nutrient solution to 5.8 for all treatments. Before the different EC treatments were applied, all seedlings were watered with nutrient of EC1.2 and pH of 5.8. When the EC treatments started, we watered the plants three times a week to maintain stable EC levels for all treatments. Each time, 3 L of different EC nutrient solutions were given in the growth containers, maintained for half an hour, and then the extra solutions were drained.

### Measurements of pakchoi fresh weight, dry weight content and leaf water content

After 20 days of the different EC treatments, shoots of each treatment were harvested for fresh weight (FW) measurement, then dried in an oven at 80°C for 5 days and reweighed for dry weight (DW). At least three replicates per pot were measured in each treatment. Leaf water content (%) was calculated as (FW-DW)/FW * 100%.

### Measurements of leaf gas exchange parameters

Leaf gas exchange was measured with Li-6400 Portable Photosynthesis System (Li-Cor Inc., Lincoln, NE, USA) on a fully developed leaf from the middle of each seedling. Measurements of photosynthesis were repeated once for each leaf, and six leaves were measured for each treatment. Irradiance level was set at 600 μmol photons m^-2^ s^-1^. CO_2_ concentration was set at 400 μmol mol^−1^ with air temperature and relative humidity set at the greenhouse conditions. Leaves were allowed to acclimate to each irradiance level for about 2 min before reading. The net photosynthetic rate (*P*_*n*_), stomatal conductance (*G*_s_), intercellular CO_2_ concentration (*C*_*i*_) and transpiration rate (*T*_r_) of pakchoi leaves were measured.

### Measurements of leaf relative chlorophyll, ascorbic acid, crude fiber, crude protein, soluble carbohydrates content and taste

Chlorophyll content was measured on the middle of intact, fully developed leaves using a chlorophyll meter SPAD-502 (Minolta, Japan) which provides a rapid, accurate and non-destructive estimate of leaf chlorophyll content. At least five leaves were measured for each treatment [19]. Ascorbic acid of leaf tissue was measured using the titration method with 2, 6-dichlorophenol-indophenol sodium salt dehydrate [20]. Crude fiber content was measured using the improved method of Śmiechowska and Dmowski [21]. The plant samples (2 g dry samples) were boiled in 200 ml 1.25% sulfuric acid solution for 30 min, and the insoluble residue was filtered and washed. The obtained substance was subsequently boiled in 200ml 1.25% potassium hydroxide solution for 30 min, filtered and washed. A sample thus prepared was dried for 2 h in the oven at 130°C. Finally, weight loss was determined after burning at 550°C. The content of crude fiber in the leaf sample was calculated in weight loss percent relative to the fresh weight of the product. Crude protein was determined according to Licitra et al. [22]. The soluble carbohydrates were determined according to Dubois et al. [23]. Tastes was determined by 5–10 persons who ate boiled plants in the different EC treatments and graded the plants from 0 (poor taste) to 10 (good taste).

### Measurements of nitrate and nitrite contents

Fresh plant leaves (2g) were added to a test tube with 10 ml distilled water. The test tube was heated in boiling water for 30 min, then cooled down with tap water. The extract in the tube was filtered into 100 ml flask, and the residue was repeatedly washed, and finally distilled water was added to the flask to make the solution of 100 ml. The filtrate was used for nitrate and nitrite content determinations. The nitrate concentration of the filtrate was detected using the method of Cataldo et al. [24]. The content of the nitrite in the filtrate was determined by the method of Kaur et al. [25].

### Antioxidant enzyme activity assay

For the enzyme assays, 0.3 g of leaf sample was ground in 3 ml of ice-cold 25 mM HEPES buffer (pH 7.8) containing 0.2 mM EDTA, 2 mM AsA, and 2% PVP. The homogenate was centrifuged at 4°C for 20 min at 12,000 *g*, and the supernatant was used for the determination of enzymatic activity. Superoxide dismutase (SOD) activity was measured in a reaction mixture containing 50 mM phosphate buffer (pH 7.8), 0.1 mM EDTA, 13 mM methionine, 75 μM nitroblue tetrazolium (NBT), 2 μM riboflavin, and 50 μl enzyme aliquot [26]. One unit of SOD activity was defined as the amount of enzyme required to cause a 50% inhibition of the rate of p-nitro blue tetrazolium chloride reduction at 560 nm. The method of Cakmak and Marschner [27], with some modifications, was used to determine the activity of guaiacol peroxidase (G-POD). The reaction mixture contained 25 mM phosphate buffer (pH 7.0), 0.05% guaiacol, 1.0 mM H_2_O_2_ and 100 μl enzyme extract. The increase in absorbance at 470 nm caused by guaiacol oxidation was used to determine the G-POD activity.

### Statistical analysis

Analysis of variance (ANOVA) was conducted using the SAS Statistical Analysis System (SAS version 9.3; SAS Institute Inc., Cary, NC). Each value was presented as the mean ± standard error (SE), with a minimum of three replicates. Differences between treatment means were tested using the Least Significant Difference (LSD) method at P ≤ 0.05 level of significance. Figures were plotted using Origin 7.5 software (Origin Lab, Northampton, MA, USA).

## Results

### Plant fresh weight, dry weight and leaf size

**Fig 1:**
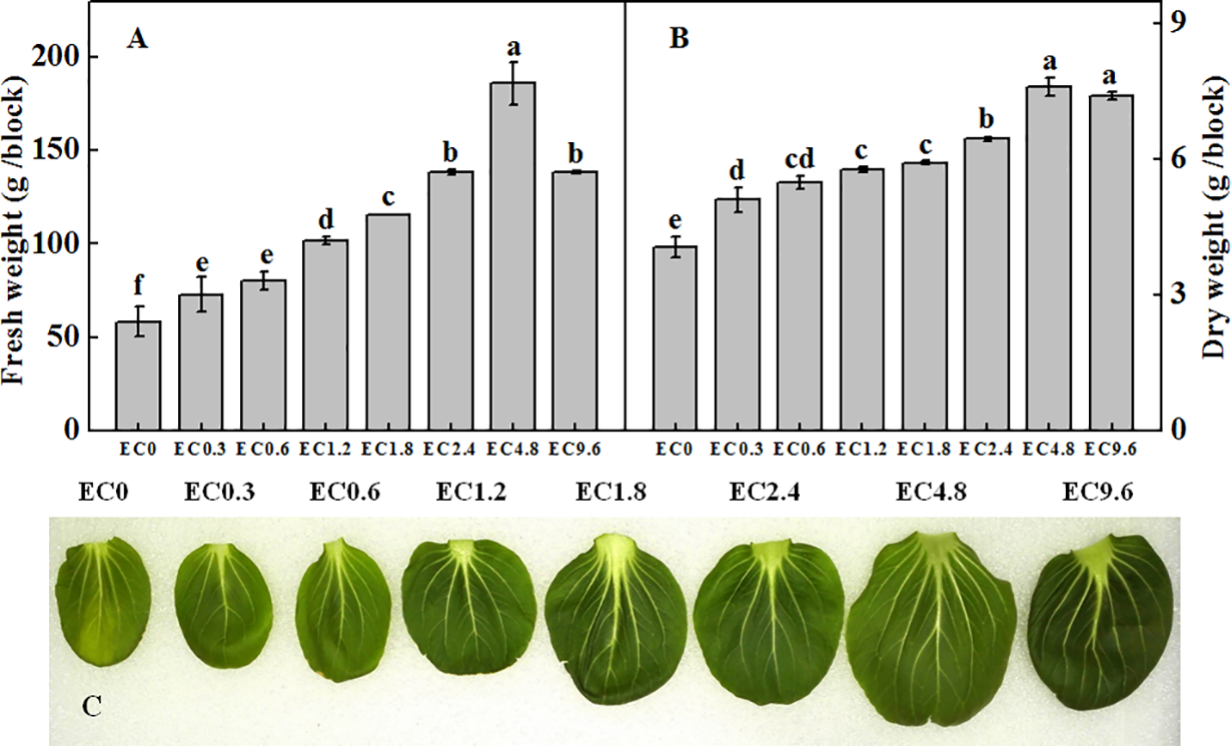
Effect of different electrical conductivity (EC) treatments on pakchoi fresh weight (A), dry weight (B) and largest leaf (C) after 20 days of treatment. Data represent mean ± SE (n = 3). Different letters indicate significant differences at p≤0.05 based on the Least Significant Difference test.

The production and leaf size of pakchoi varied in different EC treatments (Fig 1). Both FW and DW of Pakchoi leaves increased with the increase of EC level, reached the highest value in the EC4.8 treatment, and decreased in the higher EC treatments. FW in the EC9.6 treatment was similar to that in the EC2.4 treatment, but DW was significant higher than EC2.4 treatment. The leaf size showed similar pattern to the production which the largest leaf also appeared in the EC4.8 treatment.

### Leaf gas exchange parameters

**Fig 2:**
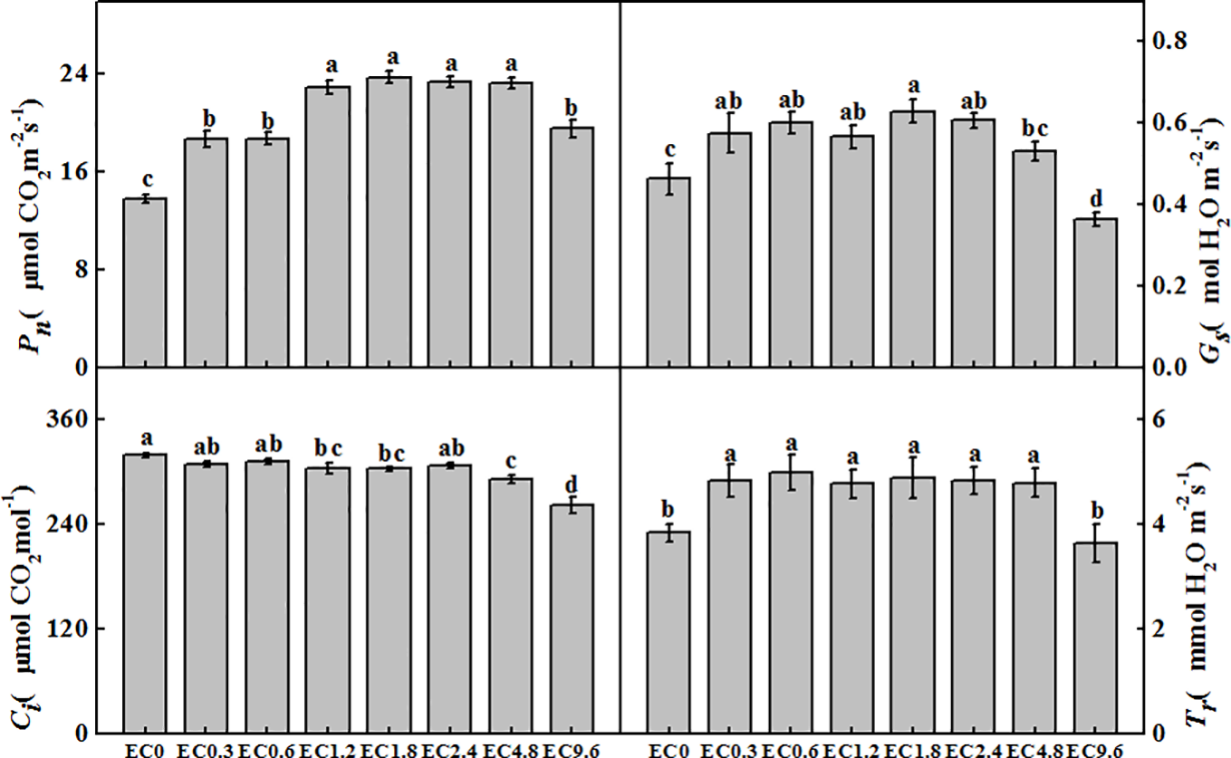
Effect of different electrical conductivity (EC) treatments on net photosynthetic rate (*P*_*n*_), stomatal conductance (*G*_*s*_), intercellular CO_2_ concentration (*C*_*i*_) and transpiration rate (*T*_*r*_) after 20 days of treatment. Data represent the mean ± SE (n = 3). Different letters indicate significant differences at p≤0.05 based on the Least Significant Difference test.

The EC treatment had significant effects on *P*_*n*_, *G*_s,_ *C*_*i*_ and *T*_r_ (Fig 2). Interestingly, there were no significant differences in *P*_*n*_ among the EC1.2, EC1.8, EC2.4, and EC4.8 treatments. But the EC0, EC0.3, EC0.6 and EC9.6 treatments showed significantly lower *P*_*n*_, with the lowest in the EC0 treatment. The changes of *G*_s_ were similar to *P*_*n*_, but there were no significant differences among the treatments from EC0.3 to EC2.4. *G*_s_ in the EC0, EC4.8 and EC9.6 treatments clearly decreased and decreased most in the EC9.6 treatment. There were no clear differences in *C*_*i*_ among the treatments from EC0 to EC2.4. But the EC4.8 and EC9.6 treatments significantly decreased *C*_*i*_. *T*_r_ in the EC0 and EC9.6 treatments was significantly reduced, but there was no significant difference among other EC treatments.

### Leaf relative chlorophyll, ascorbic acid, crude fiber, crude protein, soluble carbohydrates content and tastes

The leaf relative chlorophyll content gradually increased as nutrient EC increased, but there were no significant differences between the EC2.4 and EC4.8, between the EC1.8 and EC2.4, between the EC1.2 and EC1.8, and between the EC0.3 and EC0.6 treatments (Table 2). The leaf water and ascorbic acid contents increased from the EC0 to EC4.8 treatments, and then decreased in the EC9.6 treatment (Table 2). The contents of leaf water and ascorbic acid were significantly higher in the EC2.4 to EC9.6 treatments compared with the EC0 to EC0.6 treatments. Conversely, the soluble sugar contents decreased from the EC0 to EC9.6 treatments and there were no significant differences from the EC0.6 to EC4.8 treatments (Table 2). The crude fiber content gradually decreased from the EC0 to EC4.8 treatments, but showed a slight increase in the EC9.6 treatment (Table 2). The crude protein content increased from the EC0 to EC1.8, and then decreased in the EC2.4 and EC4.8 treatments, but there was a significant increase in the EC9.6 treatment (Table 2). The taste was ranked higher in the EC1.2 to EC2.4 treatments than that in the EC0 to EC0.3, and EC4.8 to EC9.6 treatments (Table 2).

**Table 2:** Effect of different EC treatments on relative chlorophyll content, leaf water content, ascorbic acid content, crude fiber content, crude protein content, soluble sugars and taste scores after 20 days of treatment. Data represent the mean ± SE (n = 3). Different letters indicate significant differences at p≤0.05 based on the Least Significant Difference test.

Treatments	Relative Chlorophyll	Leaf Water (%)	Ascorbic acid (mg/g)	Crude Fiber (% FW)	Crude Protein (% FW)	Soluble Sugars (% FW)	Taste Scores
EC0	23.5±1.4f	92.92±0.36d	0.57±0.03b	0.98±0.02a	1.08±0.002d	1.69±0.17a	4.36±0.69d
EC0.3	30.1±0.9e	92.98±0.22d	0.62±0.06b	0.92±0.02b	1.11±0.036c	1.13±0.08b	6.16±0.67c
EC0.6	33.4±1.2e	93.20±0.39d	0.63±0.03b	0.87±0.02c	1.13±0.001c	0.96±0.09bc	6.50±0.59bc
EC1.2	39.3±0.8d	94.36±0.16c	0.70±0.04ab	0.76±0.01d	1.13±0.006c	0.93±0.08bc	7.88±0.33ab
EC1.8	40.8±0.9cd	94.89±0.43bc	0.71±0.06ab	0.70±0.02e	1.25±0.011b	0.87±0.15bc	8.00±0.55ab
EC2.4	44.2±1.9bc	95.31±0.17ab	0.82±0.07a	0.64±0.02f	1.12±0.021c	0.82±0.07bc	8.22±0.37a
EC4.8	46.0±2.2b	95.89±0.16a	0.86±0.06a	0.53±0.02h	1.06±0.035d	0.82±0.12bc	6.16±0.50c
EC9.6	54.4±2.5a	94.59±0.18bc	0.82±0.09a	0.56±0.02g	1.36±0.015a	0.74±0.03c	4.20±0.37d

### Nitrate and nitrite contents

**Fig 3:**
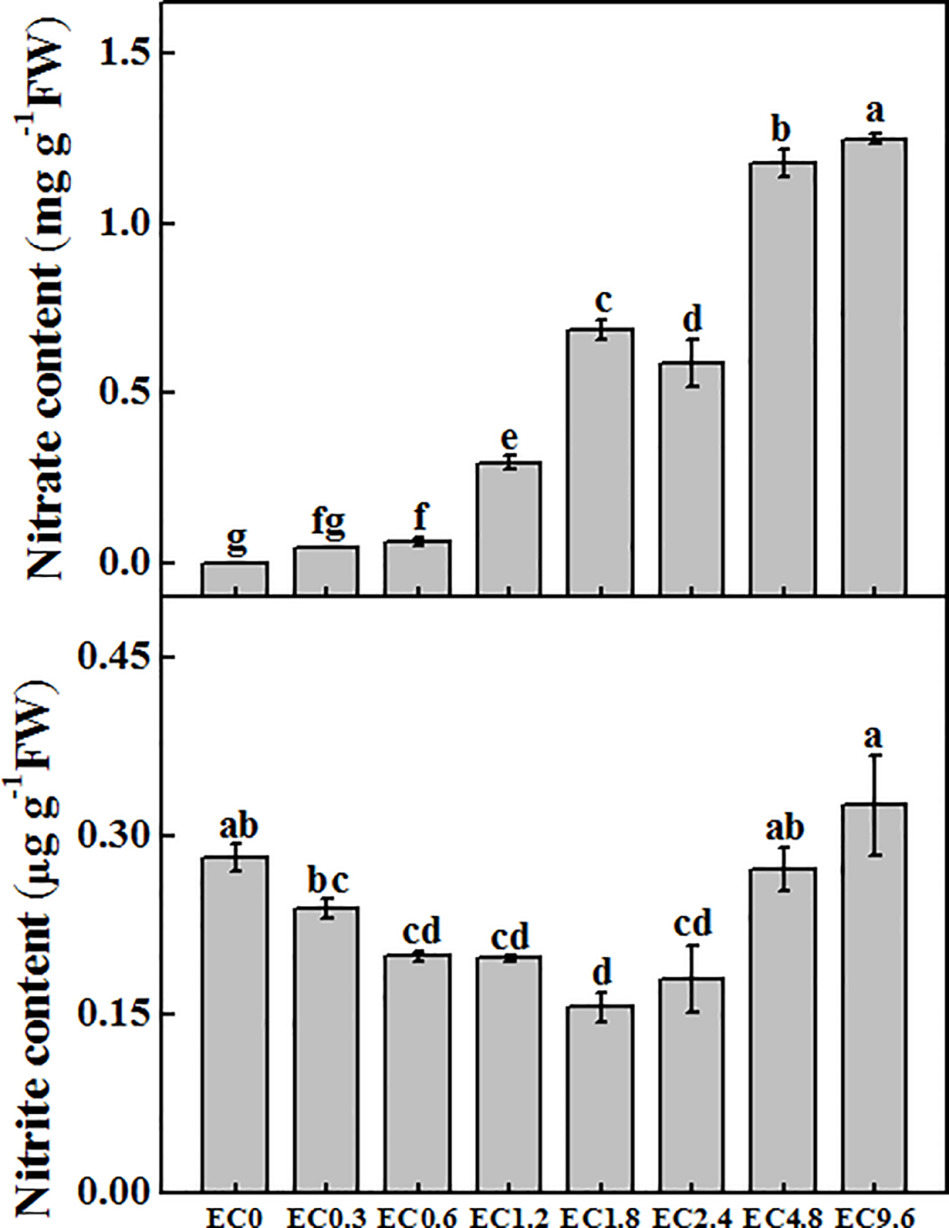
Effect of different electrical conductivity (EC) treatments on nitrate content and nitrite content after 20 days of treatment. Data represent the mean ± SE (n = 3). Different letters indicate significant differences at p≤0.05 based on the Least Significant Difference test.

The nitrate contents in the EC0, EC0.3, and EC0.6 treatments were very low and increased to 0.297 mg g^-1^ and 0.686 mg g^-1^ in the EC1.2 and EC1.8 treatments, respectively (Fig 3). The nitrate content reached 1.18 mg g^-1^ and 1.25 mg g^-1^ in the EC4.8 and EC9.6 treatments, respectively. The nitrite content was the lowest in the EC1.8 treatment and increased in the treatments with low and high EC levels (Fig 3).

### Antioxidant enzyme activity

**Fig 4:**
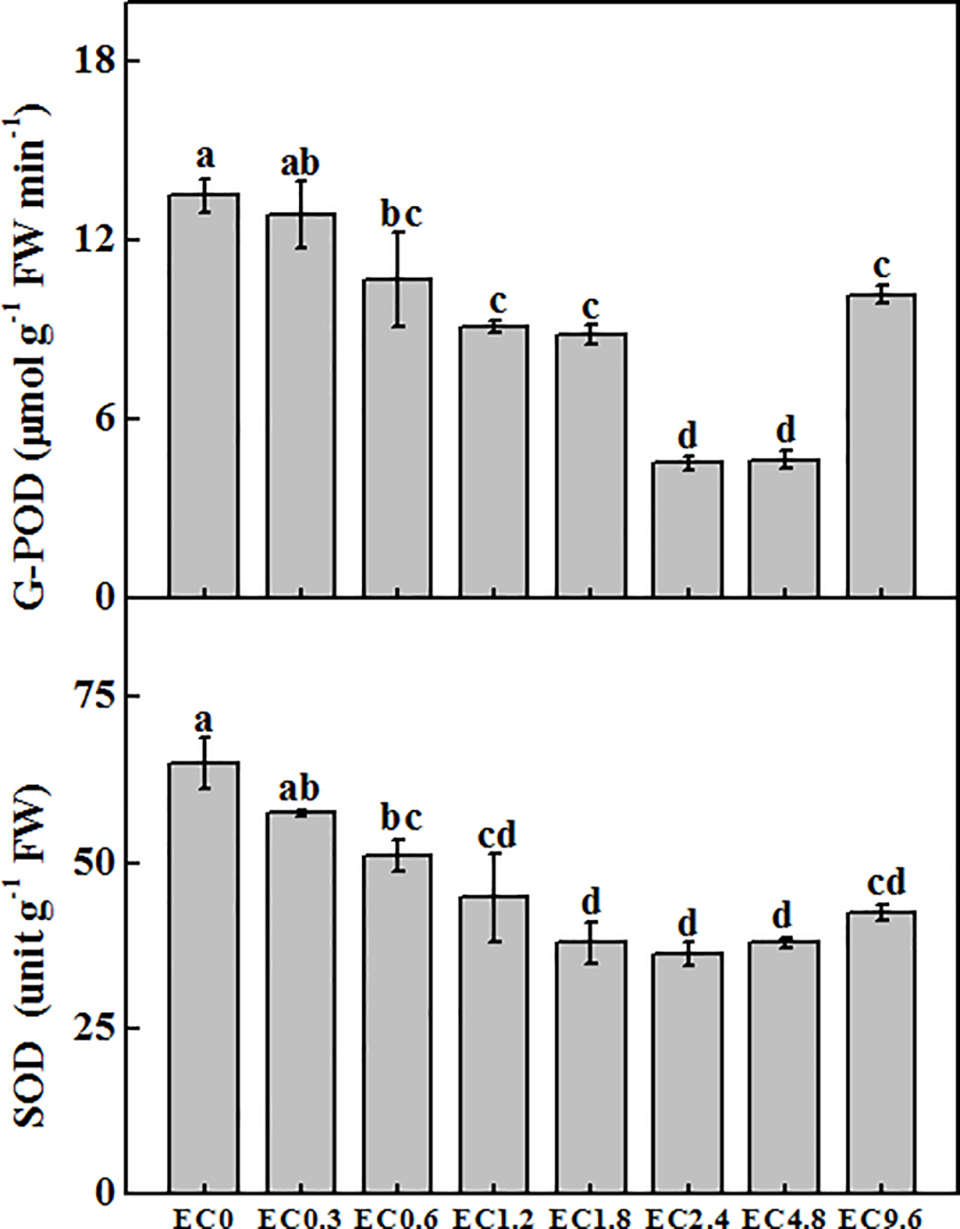
Effect of different electrical conductivity (EC) treatments on antioxidant enzyme activities of superoxide dismutase (SOD) and peroxidase (POD) after 20 days of treatment. Data represent the mean ± SE (n = 3). Different letters indicate significant differences at p≤0.05 based on the Least Significant Difference test.

Different EC treatments also induced significant changes of antioxidant enzyme activities (Fig 4). We found that G-POD significantly decreased when EC increased from the EC0 to EC2.4 treatments while there was no significant difference of G-POD between the EC2.4 and EC4.8 treatments. G-POD activities increased in the EC9.6 treatment. Similar pattern was observed for the SOD (Fig 4). Although the SOD activities were the lowest in the EC2.4 treatment, there were no significant differences among the EC1.2 to EC9.6 treatments. The highest SOD was observed in the EC0 and EC0.3 treatments.

### Plant growth

**Fig 5:**
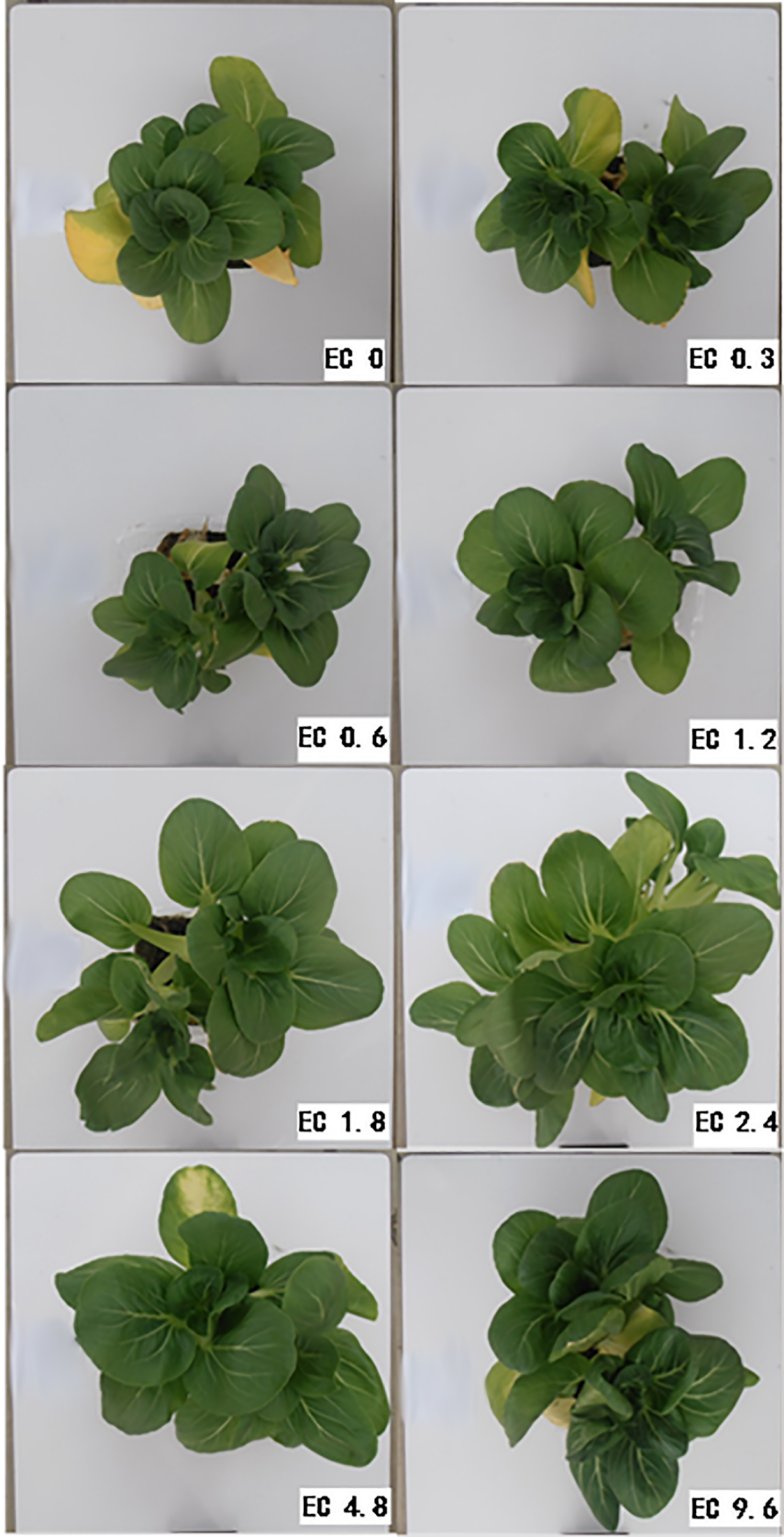
Pictures of individual plants of different electrical conductivity (EC) treatments after 20 days of treatment.

**Fig 6:**
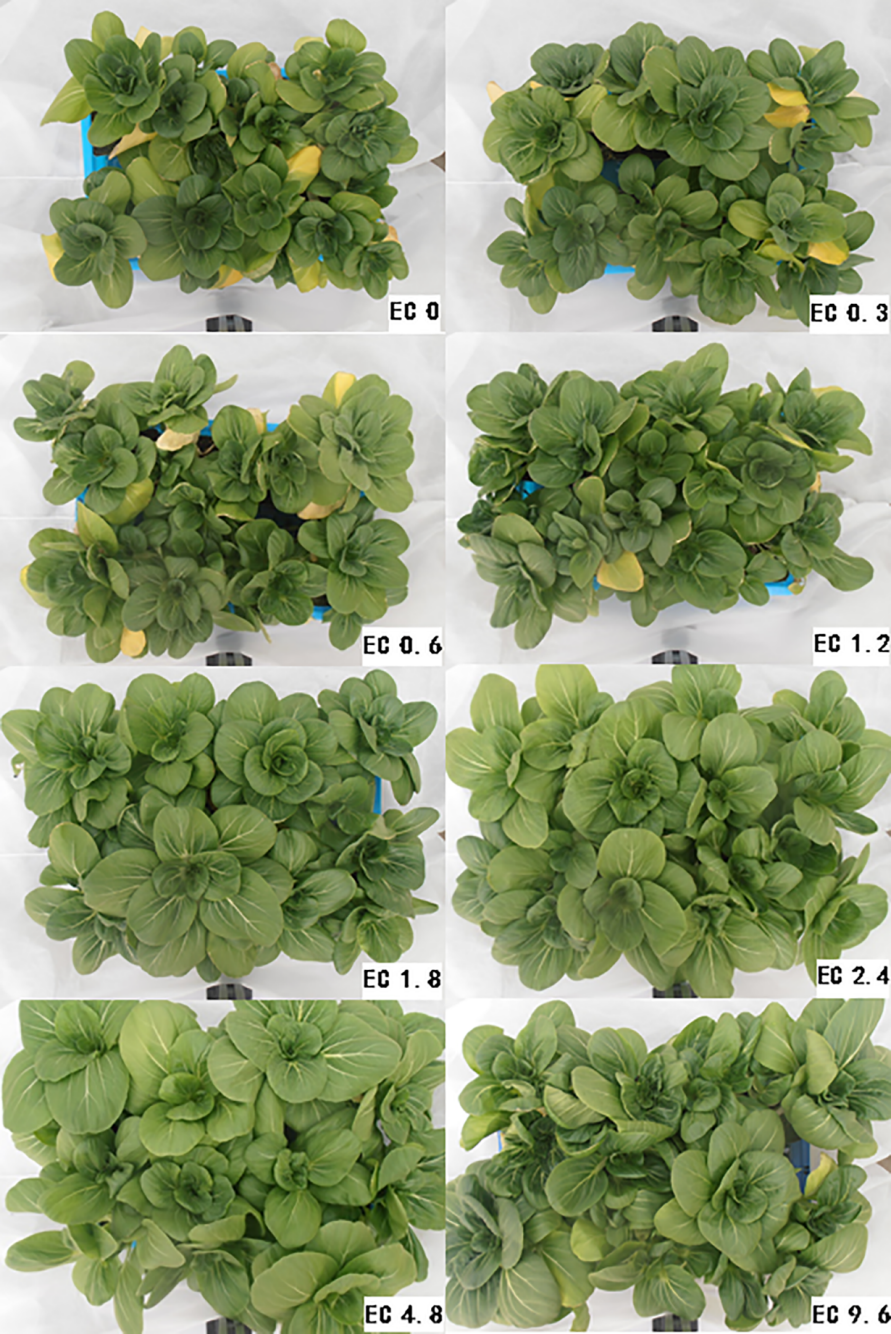
Pictures of plants in the pots of different electrical conductivity (EC) treatments after 20 days of treatment.

Plant growths of pakchoi showed clearly differences in the different treatments (Figs 5 and 6). Plants in the EC0, EC0.3, EC0.6, and EC1.2 treatments had smaller leaves, lower biomass, and some yellow leaves. The plants grew better in the treatments with EC level was 1.8 dS m^-1^ and higher. Plants in the EC1.8, EC2.4, and EC4.8 treatments appeared to have larger leaves and more biomass. In the EC9.6 treatment, plants were inhibited and had dark green color for new leaves, and yellow color for some old leaves.

## Discussion

Efficient management of nutrients is one of the main challenges for agricultural production. The elements and concentration of nutrients are important for plant growth and development [28]. In the present study we found that the fresh weight, dry weight, and leaf size of pakchoi plants gradually increased with the increase in EC, and had the highest values in the EC4.8 treatment. The highest EC9.6 treatment had lower production and leaf size due to the toxic effects of the very high nutrient solution concentration. Similar result was reported by Albornoz and Lieth [18] who found that high concentration of nutrients (EC of 6 and 10 dS m^-1^) in the root zone reduces yield of lettuce because of a combination of decreased stomatal conductance and leaf area. Samarakoon et al. [9] also found that leaf lettuce is sensitive to high EC. The plant fresh and dry weights were clearly reduced at high EC level. Leaf lettuce grows best (the highest fresh and dry weights) at EC of 1.4 dS m^-1^ under tropical greenhouse conditions (38.5°C). The differences in optimal EC levels could be caused by different leaf vegetable varieties and different growth climate conditions.

Reduced leaf photosynthesis often leads to low assimilation production [29]. In this study, we found that *P*_*n*_ and *T*_*r*_ in the low or high EC treatments was significantly decreased compared with the medium EC treatments. This could be caused by salinity stress in the high EC treatment and nutrient deficiency in the low EC treatments [3, 30, 31]. Leaf photosynthetic rates of lettuce and tomato are also affected by different nutrients management [17, 32].

Notably, we found that leaf relative chlorophyll content was significantly lower in the low EC treatments (Fig 1C and Table 2), probably due to the deficiencies of nutrient elements such as N, Mg, and Fe which were important for chlorophyll biosynthesis. Belkhodja et al. found that Fe-deficient plants had clearly inhibited the chlorophyll content increase [33]. The highest leaf chlorophyll content was found in the EC9.6 treatment. Increase in leaf chlorophyll concentration or dark color of leaves has been reported for tomato plants grown under the high EC level [34, 35]. Stefanov et al. [36] found that plants increase chlorophyll content under salt stress to enhance the salt tolerance, similar to this study as the high EC9.6 treatment induced a evidently salinity stress. The relative chlorophyll content for different EC treatments also contributed to the photosynthesis changes.

Vitamin C is one of the most important quality factors in many horticultural crops and an essential substance for humans. More than 90% of vitamin C in human diets is supplied by fruits and vegetables through ascorbic acid and dehydroascorbic acid [37]. In this study, ascorbic acid showed an increasing trend up to 2.4 dS m^-1^, but did not significantly increase over this EC level. Lisiewska and Kmiecik [38] reported that increasing amount of nitrogen fertilizer from 80 to 120 kg ha^-1^ does not affect the content of ascorbic acid in broccoli, but decreases the ascorbic acid content by 7% in cauliflower. Müller and Hippe [39] found ascorbic acid concentration is positively correlated with the nitrogen supply in butter head lettuce. This discrepancy between different vegetable crops may be due to the differences in growth habitats and growing conditions. Lee and Kader [37] revealed that plant tissues would enrich more ascorbic acid content when received higher intensity of light during the growing season. High intensity of light would support the plants get high photosynthesis which would transfer more assimilation production for plant growth and metabolism. This also coincides with our results that the changes of ascorbic acid were correlated with photosynthesis of different treatments. However, light intensity exceeding plant requirements may represent a constraint to ascorbic acid synthesis; in this respect, Conti et al. [40] found higher values in pumpkin fruits grown in greenhouse than in open field ones.

Crude fiber in plants mostly originates from cellular walls, sclerenchyma, colenchyma, and transport tissues [21]. It was observed that crude fiber decreased with EC increasing from the EC0 to EC4.8 treatment in our experiment. The highest crude fiber content was found in the control (EC0 treatment). Similar results were found by Almodares et al. [41] who reported that plants crude fiber content has a negative relationship with nitrogen fertilizer implementation. The diets with high content of fiber have been reported to have a positive effect on health, but too high crude fiber would negatively influence the food quality because high crude fiber percentage in diets will reduce digestibility [42, 43]. This is consistent with our result that the taste of pakchoi was poor in the high and low EC treatments. Pakchoi tasted better in the EC1.2 to EC2.4 treatments.

Crude protein is a term for the total protein content of a food source as determined by its nitrogen content, and is a very important component of food quality [43]. In this study, we found crude protein content increased with the increased EC treatment to 1.8 dS m^-1^. The same result was found by Mullins et al. [44] who reported that nitrogen fertilizer increased crude protein content in corn. The EC2.4 and EC4.8 treatments had clearly decreased, and EC9.6 treatment had increased the crude protein content mainly because the leaf water content was high in EC2.4 and EC4.8 treatments and relative low in EC9.6 treatment.

A high content of soluble sugars is a desirable parameter in terms of food quality [45]. In this study, soluble sugar content decreased with the EC increase, up to EC 9.6. These results are supported by Fallovo et al. [46] who reported that increasing the EC of the nutrient solution from 0.3 to 3.6 dS m^−1^ decreases the soluble sugar content of leafy lettuce. A high respiration rate of tissue vegetable in high EC treatment may reduce sugar content [47]. Photosynthate that is not fully used in the synthesis of organic compounds and sugars are accumulated where fertilizer levels are limited [48]. Conversely, Amalfitano et al. [49] recorded an increase of soluble sugar content in "Friariello" pepper fruits up to 3.8 or 4.4 dS m^-1^ depending on the sugar compound. Islam and Khan reported seasonal fluctuations would significantly affect tomato soluble sugar content and the sugar reduction due to lower enzyme activities [50]. Further studies are needed to investigate the reasons for soluble sugar content of pakchoi decreasing with EC increase in the future.

Nitrate and nitrite are nutrients found in various leafy vegetables and are also part of food preservation systems [51]. Nitrate content in pakchoi leaves were low in the low EC treatments, but increased to high level in the EC4.8 and EC9.6 treatments. Similar result was found by Fallovo et al. [46] who reported that lettuce leaf nitrate content increases in response to an increase in nutrient solution concentration. Moreover, Morano et al. [52] recorded an increase of nitrate content in basil leaves up to 2.8 dS m^-1^. The lowest nitrite content was found in the middle EC treatment (EC1.8), and the highest nitrite content in the highest and lowest EC treatments (EC9.6 and EC0) which indicated that too low or too high EC treatment can produce higher nitrite content in pakchoi leaves. Nitrate by itself is relatively non-toxic [53, 54], but approximately 5% of all ingested nitrate is converted in saliva and the gastrointestinal tract to the more toxic nitrite [55, 56]. So a low nitrite content is preferred in leafy vegetables. The Joint Expert Committee of the Food and Agriculture (JECFA) Organization of the United Nations and the European Commission’s Scientific Committee on Food (SCF) have set an acceptable daily intake (ADI) for nitrate of 0–3.7 mg kg ^−1^ bodyweight, and have proposed an ADI for nitrite only of 0–0.07 and 0–0.06 mg kg^−1^ bodyweight [57].

The activities of antioxidant enzymes are an important tool to evaluate whether the plant suffered biotic and abiotic stresses [58–60]. Here, we found that the activities of POD and SOD were increased in the low and high EC treatments while the lowest activities of POD and SOD appeared in the EC2.4 treatment. The high activities of antioxidant enzymes in the low EC treatments could be due to the nutrient deficiency that inhibiting plant leaf development and production [61–63]. The high activities in the high EC treatments were caused by toxicity and salt stress [64, 65]. Phenolic compounds with ascorbic acid are the major antioxidants and their production could be stimulated under the salt stress. Indeed, both high ascorbic acid content and high antioxidant enzyme activity were found in the EC9.6 treatment in our study. Podsedek [11] also found high ascorbic acid and antioxidant activity in *Brassica* vegetables.

## Conclusion

Eight different EC treatments were examined in our research. Pakchoi plants of Shanghaiqing grow better in the medium EC treatments (EC1.8 to EC2.4), as they show higher photosynthetic rate and production, as well as better quality. Too low EC treatments limit plant growth due to nutrient deficiency while too high EC treatments are inhibiting due to salinity stress, as plants have to enhance activities of antioxidant enzymes to adapt to the stress conditions. The findings of this study improved our mechanistic understanding of the effects of different EC nutrient solutions on pakchio plants and are useful for optimization of nutrient solutions to improve crop production and quality.
